# Revision of the genus *Placospongia* (Porifera, Demospongiae, Hadromerida, Placospongiidae) in the Indo-West Pacific

**DOI:** 10.3897/zookeys.298.1913

**Published:** 2013-05-13

**Authors:** Leontine E. Becking

**Affiliations:** 1 Naturalis Biodiversity Center, Marine Zoology Department, PO Box 9517, 2300 RA Leiden, The Netherlands; 2 IMARES - Institute for Marine Resources and Ecosystem Studies, P.O. Box 57, 1780 AB Den Helder, The Netherlands

**Keywords:** Sponge, Indonesia, marine lake, coral reef, mangrove, anchialine pool, ITS, COI

## Abstract

Species of the genus *Placospongia* are common within the tropical Indo-West Pacific, demonstrating a wide variety of colors and either branching or encrusting growth forms. A revision of Indo-West Pacific *Placospongia* was undertaken based on a redescription of the holotypes of species of *Placospongia* from the Indian Ocean and Western Pacific and an examination of an additional 103 specimens of *Placospongia* ssp. collected from Indonesia (including Vosmaer and Vernhout 1902 material), Seychelles, India, Singapore and Micronesia. One mitochondrial (COI) and one nuclear (ITS) marker were subsequently used to differentiate species. All *Placospongia* species are characterized by selenasters and tylostyles in two size classes. The combination of microsclere diversity and morphology as well as megasclere size were shown to be informative morphometric characters, supported by molecular evidence. Live coloration and growth form is shown to be unreliable for diagnoses. The study of holotypes found that *Placospongia mixta* is a valid species and that two genus transfers are necessary: *Geodinella anthosigma* is a *Placospongia* and *Placospongia labyrinthica* is a *Geodia*. A new species is also described from an anchialine pool in Indonesia, *Placospongia santodomingoae* **sp. n.**; bringing the total fauna of *Placospongia* species in the Indo-West Pacific to five: *Placospongia anthosigma*, *Placospongia carinata*, *Placospongia mixta*, *Placospongia melobesioides*, and *Placospongia santodomingoae* **sp. n.** An identification key is given. Two additional species, possibly morphologically cryptic, have been identified by molecular markers.

## Introduction

**Figure 1.**
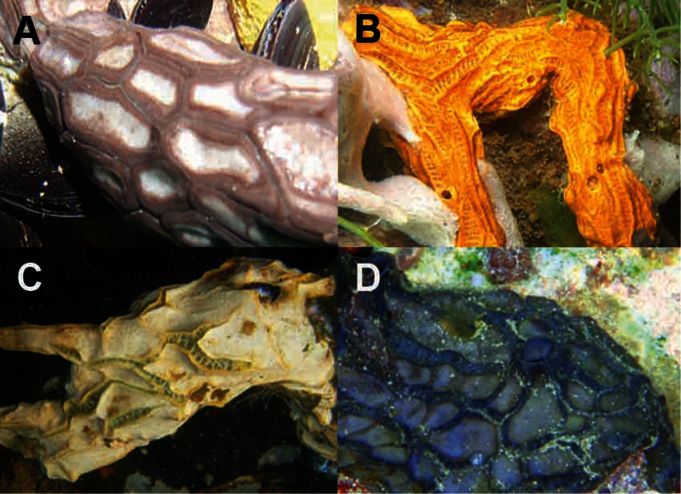
*In situ* underwater images of *Placospongia* spp. in Indonesia, displaying natural variation in color and growth form of live specimens. **A** *Placospongia mixta* (by L.E. Becking) **B** *Placospongia carinata* (by L.E. Becking) **C** *Placospongia carinata* (by L.E. Becking) **D** *Placospongia melobesioides* (by N.J. de Voogd).

**Figure 2.**
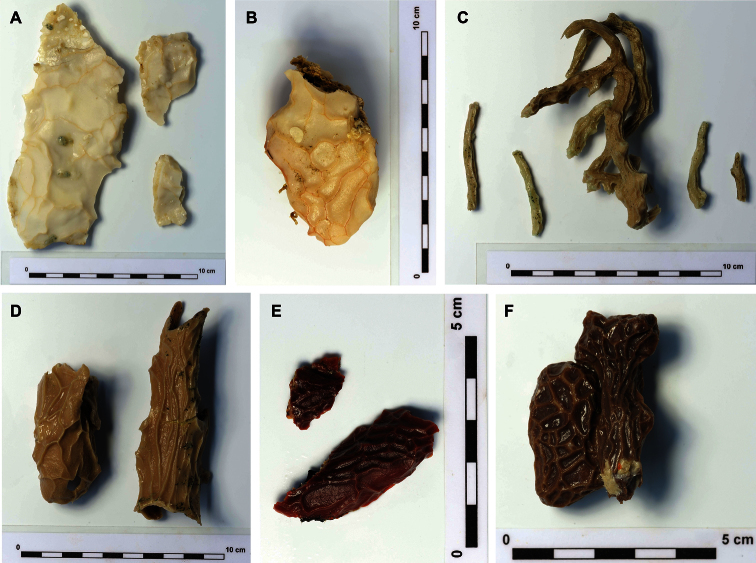
Gradation of external coloration in preserved specimens. **A** *Placospongia mixta* RMNH POR. 4492 **B** *Placospongia mixta* RMNH POR. 4113 **C** *Placospongia carinata* RMNH POR. 4482 **D** *Placospongia carinata* RMNH POR. 4483 **E** *Placospongia mixta* RMNH POR. 3979 **F** *Placospongia melobesioides* RMNH POR. 4114.

Species of the genus *Placospongia* in the tropical Indo-West Pacific occur in a wide variety of habitats such as marine lakes, coral reefs and mangroves. They may display a variety of colors and growth forms, from encrusting to branching (Figs 1, 2). Generally only two species have been recorded in species checklists within the Indo-West Pacific (e.g. Burton 1959, Hooper and Wiedenmeyer 1994, Hooper et al. 2000, Becking et al. 2006, de Voogd et al. 2008, de Voogd et al. 2009): *Placospongia melobesioides* Gray 1867, and *Placospongia carinata* (Bowerbank 1858). A recent collection of over 100 *Placospongia* specimens during fieldtrips by the author to Indonesia in 2006 (Sulawesi), 2007 (Papua), 2008, 2009 (Berau), and to Micronesia in 2010 (Yap) revealed, however, that there were more than two species present in these faunas.

The taxonomic literature records six valid species of *Placospongia* worldwide, of which there are three from the Indian Ocean and Western Pacific: *Placospongia carinata* (type locality “South Sea”, presumably in the Pacific), *Placospongia labyrinthica* Kirkpatrick 1903 (type locality East London, South Africa, Indian Ocean), *Placospongia melobesioides* (type locality Borneo, Pacific). In 1900 Thiele described the species *Placospongia mixta* from Ternate (Indonesia), which was later synonymized with *Placospongia carinata* by Vosmaer and Vernhout in 1902. Vosmaer and Vernhout (1902) based their conclusions on a review of 26 specimens collected during the Siboga expedition to Indonesia, and this collection is presently housed at the Naturalis Biodiversity Center (Leiden, The Netherlands). Subsequently, according to the World Porifera Database (van Soest et al. 2011) *Geodinella anthosigma* Tanita and Hoshino 1989 (type locality Sagami Bay, Japan) should be transferred to the genus *Placospongia*, and *Placospongia labyrinthica* should in fact be transferred to the genus *Geodia*. These suggested genus transfers have, however, not yet been published in the peer-reviewed literature. A molecular phylogeny constructed using the internal transcribed spacer region (ITS) indicated that there were nine evolutionary lineages worldwide within the genus *Placospongia* of which there were five distinct clades in the Indo-Pacific (clades C3, C4, C5, C6 & C9) that may represent five species (Nichols and Barnes 2005). The authors did not investigate the spicule morphology of the specimens in their study, therefore it is unclear which species name can be assigned to the different clades.

The objectives of the present study were to revise the genus *Placospongia* in the Indo-West Pacific by examining the holotypes of *Placospongia melobesioides*, *Placospongia carinata*, *Placospongia mixta*, as well as 103 specimens of *Placospongia* spp. that were collected from Indonesia (including the Vosmaer & Vernhout material), Singapore, Seychelles, Madagascar, and Micronesia. In order to obtain a full view of the species from the Western Pacific and Indian Ocean the holotypes of the temperate species *Geodinella anthosigma*, and *Placospongia labyrinthica* were also examined. Subsequently it was determined if growth form and color can be used as diagnostic characteristics to identify different species of *Placospongia* in the field. Finally, the aim was to provide species names to the five clades of Indo-Pacific *Placospongia* as published by Nichols and Barnes (2005) by combining their published ITS sequences from GenBank with ITS sequences from identified species of Indo-Pacific *Placospongia*.

## Material and methods

Specimens from Indonesia were collected via snorkeling in marine lakes and mangroves, and scuba diving in reefs. For a detailed description of marine lakes in Indonesia see Becking et al. (2011). Where possible material was preserved in 96% ethanol for DNA analysis, and voucher specimens were preserved in 70% ethanol and deposited in the collections of the Naturalis Biodiversity Center, Leiden, The Netherlands (RMNH POR.). Records were made on the external morphology, skeletal architecture and spicules of all material. Spicule dimensions were measured of a subset of specimens indicated in Table 1, based on 25 measurements (unless noted otherwise) and given in the text as minimum-average-maximum. The following dimensions were measured: tylostyles length × shaft width × head width; selenasters length × width; streptasters total length × ray length; spherasters diameter; rhabds length × width. Only fully developed spicules were measured. To study the skeletal architecture hand-cut perpendicular sections of the choanosome were made. The sections were air-dried, mounted in Durcupan^®^ ACM on a microscope slide, and studied under a Leica high power microscope. Spicule preparations were made by dissolving the organic tissue of a small fragment of the specimen in commercial bleach, after which the spicules were washed >10 times with distilled water and once with 96% ethanol. The spicules were air-dried on microscope slides and mounted with Durcupan^®^ ACM. The spicules were also mounted on aluminium stubs, coated with gold-palladium and studied with a Jeol Scanning Electron Microscope.

**Table 1.** Measurements of spicules of *Placospongia carinata*, *Placospongia melobesioides*, *Placospongia mixta*, and *Placospongia santodomingoae* sp. n. Sample location, growth form, color and spicule measurements provided per specimen. Spicule dimensions are based on 25 measurements and given in the text as minimum-average-maximum. Spheraster measurements in *Placospongia melobesioides* based on less than ten measurements, due to low of abundance in specimens. <br/>

			**tylostyle blunt end**			**tylostyle sharp end**			**selenaster**		**spheraster**	**streptaster**		**microrhabd**	
	**growthform**	**color live**	**length**	**max width**	**head width**	**length**	**max width**	**head width**	**length**	**width**	**diameter**	**total length**	**length ray**	**length**	**width**
*Placospongia carinata*															
R122b-86g-BK1390 (holotype)			500-**710.4**-800	10-**13.4**-15	10-**15.3**-18	140-**317.4**-450	5-**8.4**-12.5	8-**9.3**-13	80-**90**-98	60-**71.3**-85		23-**33.8**-43	8-**11.6**-15	8-**12.0**-18	2.5
RMNH POR. 4482	branching	orange	660-**726**-800	10-**12.3**-15	10-**14.5**-18	180-**263**-410	3-**5**-7.5	8-**7.5**-8	65-**71.5**-75	50-**58.5**-65		15-**34**-48	10-**13.0**-15	8-**11.7**-15	2.5
RMNH POR. 4483	encrusting	light brown	610-**703.8**-800	10-**13.1**-15	13-**14.9**-18	190-**286.7**-470	5-**6.4**-10	5-**8.6**-13	60-**80**-85	60-**62.9**-70		20-**33.7**-40	10-**13.2**-15	8-**11.9**-18	2.5
RMNH POR. 4484	encrusting	cream	560-**709.16**-920	8-**11.7**-18	10-**13.9**-18	175-**267.1**-550	3-**4.4**-10	5-**6.4**-13	50-**61.8**-70	35-**47.4**-55		25-**29.7**-35	8-**11.0**-15	10-**13.3**-18	2.5
RMNH POR. 4485	branching	dark brown	550-**761.2**-930	10-**14**-18	13-**15.5**-18	210-**295.2**-450	3-**5.6**-8	5-**7.6**-10	28-**63**-73	38-**50**-58		20-**27.6**-38	5-**9.0**-13	5-**9.4**-13	<2.5
RMNH POR. 744	encrusting	purple	450-**748.6**-980	8-**11.1**-13	10-**13.2**-15	195-**256.8**-550	5-**6.2**-10	5-**6.7**-8	60-**66.3**-70	50-**55.6**-65		25-**29.9**-38	10-12.918	8-**10.8**-13	<2.5
RMNH POR. 754	encrusting	white	540-**705.8**-830	10-**12.8**-15	13-**15.2**-18	280-**355.5**-500	5-**7.0**-10	5-**8.6**-13	55-**67.7**-75	45-**51.8**-55		25-**30.9**-38	8-**9.5**-13	8-**12.3**-18	2.5
RMNH POR. 755	encrusting	cream	560-**764.7**-910	8-**12.2**-15	10-**14.7**-18	250-**311.8**-360	5-**7.3**-8	5-**8.2**-10	55-**61.1**-65	38-**47.5**-55		30-**32.9**-38	8-**9.8**-13	8-**10.2**-13	2.5
ZMA Por. 10727	encrusting	-	620-**738.7**-840	8-**11**-13	13-**15.5**-18	240-**258.3**-270	3-**3.3**-5	3-**4.6**-8	50-**58.8**-78	35-**42.5**-63		25-**27.6**-38	8-**11.1**-15	8-**8.1**-10	<2.5
ZMA Por. 9189	branching	-	550-**703.3**-820	10-**12.8**-15	13-**15**-18	210-**318.8**-410	5-**7.5**-10	5-**9.7**-13	63-**72.2**-78	50-**56.8**-65		30-**35**-48	8-**10.7**-15	8-**9.2**-13	2.5
*Placospongia melobesioides*															
BMNH52.4.1.14 (holotype)	branching	dark brown	670-**879.6**-1010	10-**13.2**-18	10-**16.3**-20	205-**293.4**-420	5-**9.9**-13	5-**9.9**-13	58-**63.1**-68	45-**51.7**-68	15-**16.8**-18				
RMNH POR. 4495	encrusting	dark brown	480-**717.6**-1040	5-**9.5**-15	8-**10.3**-15	190-**297.6**-370	3-**5.8**-8	3-**6.1**-8	45-**56.6**-70	30-**41.6**-50					
RMNH POR. 4496	branching	dark brown	580-**778.4**-900	8-**11.7**-15	10-**14.1**-18	230-**272.8**-400	5-**7.4**-10	8-**9.1**-10	45-**60**-75	35-**45**-63					
RMNH POR. 4497	branching	dark brown	620-**745.2**-860	10-**12.2**-15	13-**14.8**-18	250-**320.8**-450	5-**8.8**-10	5-**9.4**-13	63-**70.8**-83	45-**59.6**-65					
RMNH POR. 3935	encrusting	dark brown	460-**660.9**-760	10-**11.6**-15	10-**13.7**-18	210-**325.8**-450	3-**7.4**-13	3-**8.3**-13	45-**63.9**-70	38-**51.3**-60	15-20				
RMNH POR. 3166	encrusting	dark brown	460-**704.8**-810	8-**11.4**-13	10-**13.2**-15	200-**288**-470	3-**9.5**-13	5-**10.8**-15	60-**63.6**-70	50-**50.2**-55					
RMNH POR. 3976	branching	dark brown	600-**793.6**-910	10-**12**-15	13-**14**-18	190-**321.2**-450	5-**8.5**-13	5-**9.6**-13	48-**66.8**-75	48-**55.2**-65					
RMNH POR. 3977	branching	brown	510-**683.6**-780	10-**11.5**-13	13-**13.9**-15	200-**326**-450	5-**7.5**-10	8-**9.5**-13	58-**63.3**-68	40-**46**-53					
RMNH POR. 758	branching	purple	630-**853.2**-1020	10-**13.3**-15	13-**15.8**-18	210-**253.2**-310	5-**9.5**-13	8-**11.8**-15	50-**55.2**-62.5	35-**42.3**-50	15				
RMNH POR. 757	branching	white	550-**829.2**-960	10-**13.3**-16	13-**15.8**-18	260-**302.1**-370	8-**9.6**-13	10-**11.2**-15	55-**60.4**-65	43-**48.0**-53					
RMNH POR. 2464	branching	-	710-**933.4**-1080	12.5-**15**-17.5	13-**15.7**-20	240-**326.7**-330	5-**9.2**-13	5-**10.8**-15	67.5-**81**-87.5	60-**72.5**-85					
ZMA Por. 10459	branching	brown	520-**670.8**-820	7.5-**11.4**-12.5	10-**13.4**-17.5	310-**362.5**-430	5-**8.8**-10	5-**10.1**-13	62.5-**68.9**-72.5	50-**55.5**-65					
*Placospongia mixta*															
ZMB3204 (holotype)	encrusting	-	355-**672.4**-940	8-**12.1**-18	8-**15.6**-20	165-**226.4**-275	3-**6.1**-8	3-**7.8**-10	55-**69.8**-75	43-**55.4**-73	20-**25**-30	15-**23.9**-33	3-**7.6**-13	5-**7.1**-10	<2.5
RMNH POR. 4112	encrusting	red	480-**870**-1040	10-**12.7**-15	13-**15.8**-28	210-**288**-410	5-**6.2**-10	5-**7.2**-10	50-**66.6**-75	38-**50.7**-58	18-**20.2**-25	18-**23.7**-35	5-**6.4**-10	5-**6.4**-10	<2.5
RMNH POR. 4113	encrusting	cream	550-**817.6**-1030	10-**13.1**-15	13-**15.6**-18	160-**260**-350	5-**7.3**-10	5-**8.2**-12.5	62.5-**66**-70	45-**53**-57.5	20-**22.1**-25	20-**24.8**-30	5-**5.7**-8	5-**7.5**-10	2,5
RMNH POR. 742	branching	red	550-**759.2**-850	10-**11.9**-15	10-**14.9**-20	120-**230**-380	3-**5.9**-10	3-**7.6**-10	50-**65.4**-73	33-**46.5**-56	22-**23.4**-25	15-**22.2**-35	2-**5.7**-8	5-**7.4**-10	<2.5
RMNH POR. 4489	encrusting	cream	630-**886.6**-1010	10-**12.9**-15	13-**15.4**-19	175-**221.5**-320	3-**3.9**-8	2-**7.2**-10	60-**68**-75	43-**50.8**-58	18-**20.6**-25	20-**26.1**-35	8-**10.8**-15	8-**8.5**-10	<2.5
RMNH POR. 4490	encrusting	cream	510-**727.6**-970	8-13.120	13-**16.3**-23	150-**240**-310	3-**5.3**-8	2-**6.4**-8	55-**70.4**-83	40-**53.3**-65	13-**20.5**-25	15-**21.7**-30	5-**6.4**-13	8-**9.2**-13	<2.5
RMNH POR. 4491	encrusting	brown	780-**1001.4**-1200	10-**14.8**-18	15-**17.5**-20	240-**284**-350	5-**6.3**-8	5-**8.3**-10	60-**71**-75	48-**57.5**-63	18-**23**-25	20-**27.3**-35	5-**7**-10	5-**6.3**-8	2,5
RMNH POR. 4492	encrusting	white	610-**995.8**-1250	10-**16**-20	13-**19**-25	260-**274**-290	8-**9**-10	8-**9**-10	58-**71**-78	45-**54.6**-70	15-**20.2**-25	18-**24.8**-33	10-**11.2**-15	5-**8.6**-18	<2.5
RMNH POR. 3158	encrusting	cream	550-**990**-1210	13-**16.9**-20	13-**17.5**-20	130-**267.8**-400	5-**8.8**-15	8-**9**-10	65-**71**-75	50-**56.5**-63	23-**23.8**-25	23-**28.4**-35	5-**8.7**-13	5-**6.6**-8	<2.5
RMNH POR. 745	encrusting	red	760-**914.1**-1030	13-**17**-23	10-**18**-25	250-**366.6**-480	3-**8**-13	3-**9**-13	45-**73.6**-80	45-**60**-70	20-**23.9**-25	20-**23.7**-30	3-**6.4**-9	5-**7.5**-10	<2.5
RMNH POR. 4493	encrusting	brown	460-**761.6**-1070	10-**14.6**-23	13-**17.38**-25	220-**323.6**-430	8-**9.1**-13	10-**11.3**-15	73-**80.3**-85	53-**65.3**-73	20-**26.5**-30	18-**23.4**-30	15-**8.1**-10	8-**8.7**-13	<2.5
RMNH POR. 4494	encrusting	brown	540-**758**-900	10-**12.2**-18	10-**13.8**-20	180-**216.9**-350	3-**3.3**-5	4-**4.4**-8	50-**59.1**-68	35-**42.3**-58	15-**20.9**-28	23-**26.9**-30	8-**10.4**-13	8-**8.5**-10	<2.5
*Placospongia santodomingoae* sp.n.															
RMNH POR. 4486 (holotype)	branching	brown	430-**605.6**-660	13-**15.5**-20	13-**18.1**-23	240-**261.3**-290	5-**7.2**-8	5-**8.8**-10	80-**84.8**-90	60-**67.3**-75				8-**12.3**-18	2.5-**2.7**-3.5
RMNH POR. 4487	branching	orange	530-**652.4**-740	13-**16**-20	15-**18.0**-23	220-**274.7**-310	5-**8.2**-13	8-**9.5**-15	63-**82.9**-93	60-**66.3**-73				5-**10.5**-20	2.5-**2.6**-3.5
RMNH POR. 4488	branching	orange	480-**633.2**-760	15-**17.2**-20	18-**19.6**-23	190-**273.2**-380	5-**7.9**-10	8-**10.3**-13	80-**87**-93	58-**69**-75				8-**13.5**-18	2.5-**2.9**-3.5

DNA extractions were made with Qiagen DNEasy animal blood and tissue extraction kit following the manufacturer’s protocol. The polymerase chain reaction (PCR) reaction volume was 25 *μ* l and contained 5 *μ* l Phire ® Hot Start reaction buffer, 1 unit Hotstart Phire® Hot Start DNA polymerase (Finnzymes), 2 *μ* l 1 mM dNTPs (Gibco), 1 *μ* l DNA template (5-20 ng) and 0.625 *μ* l of 10mM each primer. The standard DNA-barcoding fragment of the mitochondrial cytochrome oxidase subunit I (COI) fragment was amplified by using a specific forward primer designed by the author for *Placospongia* P-COI-F: GCA GG ATG ATA GGA ACA GGW TTT AG and the degenerated reverse primer from Folmer et al. (1994) designed by Meyer et al. (2005): dgHCO2198: TAA ACT TCA GGG TGA CCA AAR AAY CA. Temperature regime: 94°C for 30s; followed by 35 cycles of 94°C for 5s; 50°C for 5s; 72°C for 12 s; followed by 71°C for 1 min). ITS was amplified with primers from Wörheide (1998) RA2: GTC CCT GCC CTT TGT ACA CA and ITS2.2: CCT GGT TAG TTT CTT TTC CTC CGC). PCR products were purified and sequenced by Macrogen Inc (Korea and The Netherlands). The poriferan origin of the obtained sequences was verified through BLAST searches (http://blast.ncbi.nlm.nih.gov/Blast.cgi). Sequences were handled in SEQUENCHER 4.10.1 (Gene Codes Corporation) and aligned with CLUSTALW and MUSCLE implemented in DAMBE (Xia and Xie 2001). Species of the family Spirastrellidae were selected as outgroup for the phylogenetic analyses. For the COI genetree four specimens of *Spirastrella* aff. *decumbens* (RMNH POR. 4505, 4589, 4614) were taken. For the ITS genetree sequences of species from Spirastrellidae were taken from GenBank, as well as ITS sequences of Indo-Pacific *Placospongia* spp. from the study by Nichols & Barnes (2005), for GenBank accession numbers see Figure 11. The best-fit DNA substitution model was selected by the Akaike Information Criterion deployed in jMODELTEST v. 0.1.1 (Posada 2008) and this model (HKY for COI and GTR+G+I for ITS) was used for subsequent Bayesian and maximum likelihood phylogeny inferences. Phylogenetic reconstructions were performed under Bayesian inference criteria implemented in MrBayes v. 3.1.2. (Huelsenbeck and Ronquist 2001). Each analysis comprised two independent runs of four Metropolis-coupled Markov-chains, sampled at every 1000^th^ generation at the default temperature (0.2). Analyses were terminated after the chains converged significantly as indicated by an average standard deviation of split frequencies <0.001. Convergence was also checked in Tracer v. 1.5.0 (Rambaut and Drummond 2007). For comparison, maximum likelihood bootstrap analyses were conducted using MEGA v. 5.01 (Tamura et al. 2011) using a heuristic search with 1000 bootstrap replicates. Within-group and between-group uncorrected *p-* distances were calculated in MEGA.

Abbreviations used in this manuscript: Naturalis Biodiversity Center, Leiden, The Netherlands (RMNH POR.), the Zoological Museum of the University of Amsterdam (ZMA Por.), Zoologisches Museum für Naturkunde an der Universität Humboldt zu Berlin, Berlin, Germany (ZMB), The Natural History Museum, London, United Kingdom (BMNH).

## Taxonomy

### Phylum Porifera Grant, 1836 Class Demospongiae Sollas, 1885 Order Hadromerida Topsent, 1894 Family Placospongiidae Gray, 1867 Genus *Placospongia* Gray, 1867

#### Placospongia

Gray, 1867

http://species-id.net/wiki/Placospongia

##### Type species:

*Placospongia melobesioides* Gray, 1867 by monotypy

##### Description, amended from Systema Porifera (Hooper and van Soest 2002).

Encrusting to branching growth forms. Small encrustations of 3 cm^2^ to large surfaces of >2m^2^ to branching individual with total size of up to 45cm in length and branch diameter between 0.25-1.5cm. Total size of specimens is hard to establish as parts of the body may be encrusting within cracks. Dried material is hard, alcohol preserved and live specimens remain compressible as the choanosome is of more pliant material than the cortex. The surface is made up of smooth cortical plates separated by contractible grooves which form a kind of network on the surface while these are firmly closed in preserved specimens. See Vosmaer and Vernhout (1902) and Rützler (2002) for an extensive description of the genus. In live specimens grooves are open and oscules are visible inside contractile ridges, running between plates. Live color white, cream, orange, reddish brown to dark black-brown (Fig. 1, 2) and come color is usually retained after alcohol preservation. The contact lines between the plates ridge up slightly and are generally a different shade of the color of the plates.

**Skeleton.** The cortical plates consist of densely packed selenasters and can also contain auxiliary microscleres. Developmental stages of selenasters occur throughout the choanosome. Tylostyle tracts support the margins of the cortical plates. In branching specimens radial tylostyle tracts run from the centre core (consisting of densely packed selenaster) to the cortical plates, in encrusting specimens tracts run in direction from substrate to cortex. The sharp ends of the smaller tylostyles are projected beyond the cortex surface. Microscleres occur in the cortex and scattered in choanosomal skeleton. For a detailed description of external morphology and anatomy see Vosmaer and Vernhout (1902).

**Spicules.** Megascleres are tylostyles in two size classes, microscleres are selenasters, and can include choanosomal and ectosomal spirasters (slender-spined streptasters and acanthose microrhabds), spherasters, and/or spherules. Selenasters often remain pigmented after treatment with bleach or nitric acid.

#### Placospongia anthosigma

(Tanita & Hoshino, 1989)

http://species-id.net/wiki/Placospongia_anthosigma

Figure 3

Geodinella anthosigma Tanita & Hoshino, 1989: fig. 16, Plate III fig. 1

##### Material examined.

**Holotype.** NSMT-Po R288 (National Museum of Nature and Science, Tokyo, Japan), Japan, Kannonzuka-dashi, Amadaiba, Sagami Bay, 62–67m. depth.

##### Description.

**Figure 3.**
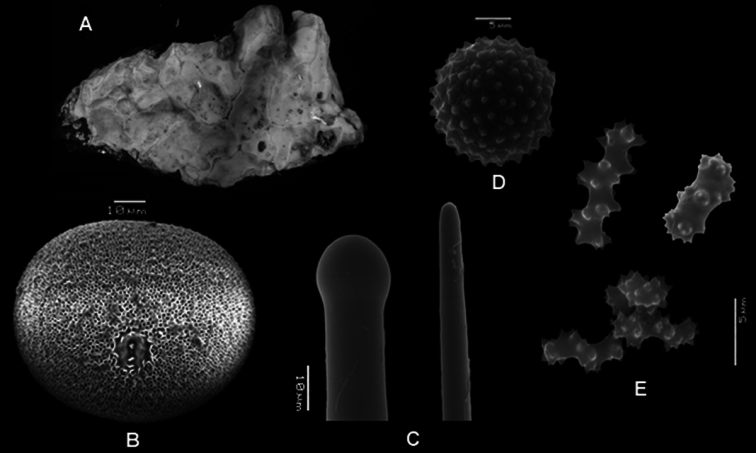
*Placospongia anthosigma* holotype (NSMT-Po R288) **A** type specimen (image taken from website database of the Museum of Nature and Science, Tokyo, Japan) **B** selenaster **C** large tylostyle (head and blunt end) **D** spheraster **E** spirasters referred to as ‘anthosigma’ by Tanita & Hoshino (1989).

HolotypeNSMT-Po R288 encrusting specimen in three pieces of 1–2cm^2^ and 5mm thick, beige to pink in alcohol (Figure 3A).

**Spicules.** Megascleres large tylostyles with blunt point 520-797-930 × 15-18-20 × 18-20-23 *μ* m, small tylostyles with blunt point 250-320-410 × 10-12-18 × 13-14-18 *μ* m; microscleres selenasters 85-90-98 × 70-73-80 *μ* m,spherasters 15-19-25 *μ* m, stout spirasters with two or three contortions and acanthose spines spirally placed on shaft 8-11-18 × 3-4.5-5 *μ* m (Fig. 3)

**Skeleton.** As description of genus with addition that spirasters form a layer over and amidst the selenaster cortex and are also prevalent in choanosomal tissue. Spherasters amidst selenaster cortex and dispersed in choanosome.

##### Distribution.

Typelocality Sagami Bay, Eastern Japan, presently not recorded from any other locality.

##### Ecology.

On rock substrate in deep temperate waters.

##### Remarks.

Originally described by Tanita and Hoshino (1989) as *Geodinella anthosigma*. *Geodinella* is no longer a valid genus. *Geodinella anthosigma* should be transferred to the genus *Placospongia* based on the external morphology with the characteristic cortical plates and the presence of selenasters, tylostyles and spherasters. *Placospongia anthosigma* is distinguished from the other Indo-Pacific *Placospongia* spp. by the presence of contorted, spirally ornamented spirasters referred to by Tanita and Hoshino (1989) as ‘anthosigma’ and the small class of tylostyles with blunt points.

#### Placospongia carinata

(Bowerbank, 1858)

http://species-id.net/wiki/Placospongia_carinata

Figures 4 , 5

**Figure 4.**
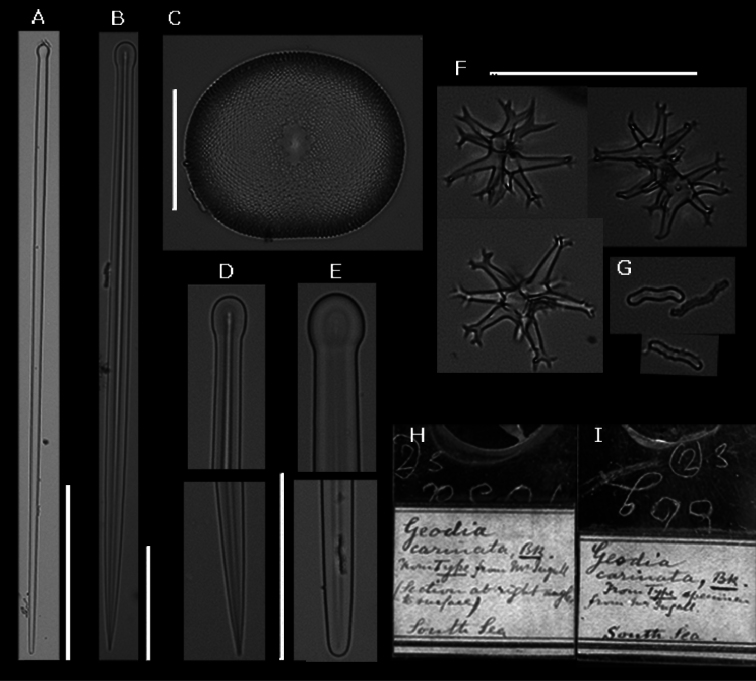
*Placospongia carinata* slide of holotype (BMNH, R1228, 86g, Bk.1390; R1275, PE01, Bk1390). **A** large tylostyle (scale=200 *μ* m) **B** small tylostyle (scale=50 *μ* m) **C** selenaster (scale=50 *μ* m) **D** close up of large tylostyle (scale=50 *μ* m) **E** close up of small tylostyle **F** streptasters (scale=50 *μ* m) **G** acanthose microrhabds **H** original slide of thick section of holotype **I** original slide of spicules of holotype.

**Figure 5.**
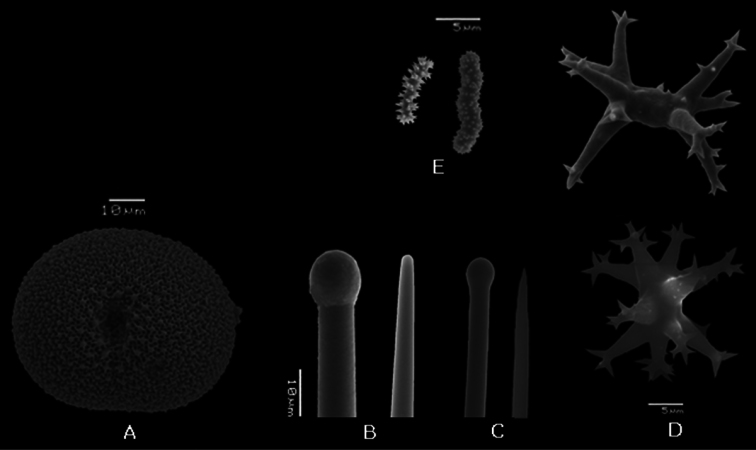
*Placospongia carinata* (RMNH POR. 4483). **A** selenaster **B** large tylostyle (head and blunt end) **C** small tylostyle (head and hastate end) **D** streptasters, E. acanthose microrhabds.

Geodia carinata Bowerbank, 1858: plate XXV fig. 19.
Geodia carinata Bowerbank, 1874: plate XLVI figs 1–5.

##### Material examined.

**Holotype.** “South Sea”: BMNH R1228 - 86g - Bk.1390 (slide), R1275 - PE01 - Bk1390 (slide).

**Vosmaer & Vernhout (1902), Siboga expedition**: RMNH POR. 755; RMNH POR. 754; RMNH POR. 744. **Other material:** RMNH POR. 4484, RMNH POR. 3943, RMNH POR. 3944, RMNH POR. 4485, RMNH POR. 3945, RMNH POR. 3946, RMNH POR. 3947, RMNH POR. 3948, RMNH POR. 3949, RMNH POR. 3950; RMNH POR. 3951, RMNH POR. 3952, RMNH POR. 3953, RMNH POR. 3954, RMNH POR. 3955, RMNH POR. 4482, RMNH POR. 3956, RMNH POR. 3957, RMNH POR. 4483, RMNH POR. 3958; ZMA Por. 8813ZMA Por. 09578; ZMA Por. 11367, ZMA Por. 16584, ZMA Por. 10727, ZMA Por. 1818, ZMA Por. 10481, ZMA Por. 20735; ZMA POR.9189. (See Table 2 for full details per specimen)

**Table 2.** Location details of reviewed specimens of *Placospongia carinata*.

**registration number**	**fieldcode**	**country**	**province**	**region**	**island**	**locality**	**habitat**	**latitude**	**longitude**	**depth (m.)**	**date**	**collector**
RMNH POR. 744	#1500	Indonesia	Moluccas	W of Aru	Kur		benthic hard			20-40	6.xii.1899	Siboga expedition
RMNH POR. 754	#1458	Philippines		Sulu Sea	Ubian islands	anchorage off North Ubian	lithothamnion	06°7.5'N, 120°26'E		23	28.vi.1899	Siboga expedition
RMNH POR. 755	#1848	Indonesia	West Papua	Raja Ampat	Misool		sand, stones	02°28'.5S, 131°3'.3E		32	20.viii.1899	Siboga expedition
RMNH POR. 3943	#KKB/mol716	Indonesia	East Kalimantan	Berau	Kakaban	Kakaban lake	marine lake	02°08'57.3"N, 118°31'26.4"E		0-2	ix.2008	L.E.Becking
RMNH POR. 3944	#KKB/mol754	Indonesia	East Kalimantan	Berau	Kakaban	Kakaban lake	marine lake	02°08'57.3"N, 118°31'26.4"E		0-2	ix.2008	L.E.Becking
RMNH POR. 3945	#KKB/mol780	Indonesia	East Kalimantan	Berau	Kakaban	Kakaban lake	marine lake	02°08'57.3"N, 118°31'26.4"E		0-2	ix.2008	L.E.Becking
RMNH POR. 3946	#KKB/mol810	Indonesia	East Kalimantan	Berau	Kakaban	Kakaban lake	marine lake	02°08'57.3"N, 118°31'26.4"E		0-2	ix.2008	L.E.Becking
RMNH POR. 3947	#KKB/mol814	Indonesia	East Kalimantan	Berau	Kakaban	Kakaban lake	marine lake	02°08'57.3"N, 118°31'26.4"E		0-2	ix.2008	L.E.Becking
RMNH POR. 3948	#KKB/mol825	Indonesia	East Kalimantan	Berau	Kakaban	Kakaban lake	marine lake	02°08'57.3"N, 118°31'26.4"E		0-2	ix.2008	L.E.Becking
RMNH POR. 3949	#KKB/mol713	Indonesia	East Kalimantan	Berau	Kakaban	Kakaban lake	marine lake	02°08'57.3"N, 118°31'26.4"E		0-2	ix.2008	L.E.Becking
RMNH POR. 3950	#KKB/mol1068	Indonesia	East Kalimantan	Berau	Kakaban	Kakaban lake	marine lake	02°08'57.3"N, 118°31'26.4"E		0-2	ix.2008	L.E.Becking
RMNH POR. 3951	#MA/mol700	Indonesia	East Kalimantan	Berau	Maratua	Haji Buang lake	marine lake	02°12'31.2"N, 118°35'46.8"E		0-2	ix.2008	L.E.Becking
RMNH POR. 3952	#MA/mol975	Indonesia	East Kalimantan	Berau	Maratua	Haji Buang lake	marine lake	02°12'31.2"N, 118°35'46.8"E		0-2	ix.2008	L.E.Becking
RMNH POR. 3953	#MA/mol947	Indonesia	East Kalimantan	Berau	Maratua	Haji Buang lake	marine lake	02°12'31.2"N, 118°35'46.8"E		0-2	ix.2008	L.E.Becking
RMNH POR. 3954	#MA/mol1055	Indonesia	East Kalimantan	Berau	Maratua	Haji Buang lake	marine lake	02°12'31.2"N, 118°35'46.8"E		0-2	ix.2008	L.E.Becking
RMNH POR. 3955	#MA/mol1012	Indonesia	East Kalimantan	Berau	Maratua	Haji Buang lake	marine lake	02°12'31.2"N, 118°35'46.8"E		0-2	ix.2008	L.E.Becking
RMNH POR. 3956	#MA/mol1001	Indonesia	East Kalimantan	Berau	Maratua	Haji Buang lake	marine lake	02°12'31.2"N, 118°35'46.8"E		0-2	ix.2008	L.E.Becking
RMNH POR. 3957	#MA/mol1009	Indonesia	East Kalimantan	Berau	Maratua	Haji Buang lake	marine lake	02°12'31.2"N, 118°35'46.8"E		0-2	ix.2008	L.E.Becking
RMNH POR. 3958	#MA/mol1500	Indonesia	East Kalimantan	Berau	Maratua	Haji Buang lake	marine lake	02°12'31.2"N, 118°35'46.8"E		0-2	ix.2008	L.E.Becking
RMNH POR. 4482	#MA/mol1061	Indonesia	East Kalimantan	Berau	Maratua	Haji Buang lake	marine lake	02°12'31.2"N, 118°35'46.8"E		0-2	ix.2008	L.E.Becking
RMNH POR. 4483	#MA/LE172	Indonesia	East Kalimantan	Berau	Maratua	Haji Buang lake	marine lake	02°12'31.2"N, 118°35'46.8"E		0-2	ix.2008	L.E.Becking
RMNH POR. 4484	#KKB/mol110	Indonesia	East Kalimantan	Berau	Kakaban	Kakaban lake	marine lake	02°08'57.3"N, 118°31'26.4"E		0-2	ix.2008	L.E.Becking
RMNH POR. 4485	#KKB/mol763	Indonesia	East Kalimantan	Berau	Kakaban	Kakaban lake	marine lake	02°08'57.3"N, 118°31'26.4"E		0-2	ix.2008	L.E.Becking
ZMA Por. 1818		Indonesia	Maluku		Banda islands	Banda anchorage	reef	04°32'23.3"S, 129°54'28.8"E		9-45	22.xi.1899	Siboga expedition
ZMA Por. 9578		Singapore			Pulau Salu		reef	01°12'59.0"N, 103°42'25.2"E		2	22.xii.1977	H. Moll
ZMA Por. 8813		Indonesia	Nusa Tenggara		Komodo	NE cape	reef	08°28'60.0"S, 119°34'4.8"E		30	19.ix.1984	R.W.M. van Soest (Snellius II Expedition)
ZMA Por. 9189		India		Laccadive Islands	Agatti					20-25	1987	National Institute of Oceanography
ZMA Por. 10481		Seychelles		Mahé	Mahé	SE coast, near Pointe Cocos	reef			35-45	24.xii.1992	R.W.M. van Soest
ZMA Por. 10727		Seychelles		Mahé	Mahé	NE Point	reef	04°34'59.9"S, 055°28'0.1"E		1	14.xii.1992	R.W.M. van Soest
ZMA Por. 11367		Seychelles		Mahé	N of Aride		reef	04°10'59.9"S, 055°40'0.1"E		40	19.xii.1992	R.W.M. van Soest
ZMA Por. 16584		Seychelles		Mahé	Mahé	SW coast, Baie Lazare, Anse Gaulettes	reef	04°10'59.9"S, 055°40'0.1"E		1-4	6.xii.1992	R.W.M. van Soest
ZMA Por. 20735		Seychelles		Mahé			reef				1992	R.W.M. van Soest

##### Description.

Reviewed material is encrusting and/or branching. External morphology follows the description of the genus. Color of live specimens can be purple brown, chocolate brown, milk coffee brown, orange brown, orange, cream, or white (Fig. 1, 2). Color of choanosome is pale beige. After preservation in ethanol specimens retain some color of the live coloration.

**Spicules.** Holotype slide with spicules R1228-86g-Bk.1390 (BMNH) and slide with thick section R1275-PE01-Bk1390 (BMNH) (Fig. 4): megascleres large straight tylostyles with blunt ends 500-**7** 10-820 × 10-13-15 × 10-15-18 μm, small straight tylostyles with sharp ends 140-317-450 × 5-8-25 × 8-9-13 μm; microscleres selenasters 80-90-98 μm, streptasters with varying number of (spined) rays (5-10) with bifurcating endings or tufts 23-34-43 × 8-15 μm, acantho microrhabds 8-12-18 × 1-2.5 μm, spherasters absent. The range within the examined material (Table 1  & Fig. 5): megascleres large tylostyles 540-990 × 8-18 × 10-18 *μ* m, small tylostyles 175-550 × 3-10 × 3-13 *μ* m; microscleres selenasters 50-85 × 35-70 *μ* m, streptasters 15-48 × 5-18 *μ* m, acanthose microrhabds 5-18 × 1-2.5 *μ* m, spherasters absent.

**Skeleton.** As description of genus with addition that microrhabds form a layer over and amidst the selenaster cortex and are also prevalent in choanosomal tissue. Spirasters scattered in choanosome.

##### Distribution.

East African coast to eastern Indonesia (Fig. 9, Table 2). Originally described from the ‘South Sea’, presumably the South Pacific Ocean. This has been interpreted by some (Rützler 2002, van Soest et al. 2011) to be Palau or Vanuatu, but this remains speculative. Based on the reviewed material and literature the minimal distribution is from Madagascar (Lévi 1956), to the Seychelles, and across Indonesia to the Aru Islands (Table 2). Distribution may extend further East.

##### Ecology.

Depth 0–45m. In Indonesia rarely found in reef environment, but high abundance in marine lakes. Possibly higher prevalence in reefs in Eastern Africa, based on the ZMA Por. collection from the Seychelles and the publication from Madagascar (Lévi 1956).

##### Remarks.

The Bowerbank description from 1858 should be considered as the original description of ‘*Geodia carinata’*, now accepted as *Placospongia carinata*,with plates XXV fig. 19 and XXVI fig. 10 representing the streptasters (“arborescent elongo-subsphero-stella”). Subsequently in 1874 Bowerbank published a more extensive description of “*Geodia carinata”* including a drawing of the streptasters (fig. 3, p.299) and spined microrhabds (“minute multiangulated cylindrical retentive spicula”, fig. 2, p.299) that he described as characteristic of the species. In neither publication registration numbers were provided, however. The habitus drawing in fig. 5, p.299 of Bowerbank publication in 1874 is identical to the specimen BMNH95.6.7.1 that I received from the BMNH after requesting the holotype for *Placospongia carinata*. In addition, I received the slides of spicules (codes: R1228, 86g, Bk.1390) and of the thick cut (codes: R1275, PE01, Bk1390) that were labeled to belong to the holotype (Fig. 5). Upon inspection I discovered that the specimen BMNH 95.6.7.1 is in fact a *Placospongia melobesioides*, while the two slides do indeed represent *Placospongia carinata* containing the characteristic streptasters with bifurcating endings and the microrhabds as indicated in the Bowerbank images and in the images taken from these slides in Fig. 5. The slides clearly do not come from the specimen BMNH 95.6.7.1. In the 16 years between Bowerbank’s 1858 and 1874 publications, I fear that there has been some exchange or misinterpretation of the labels of the specimens resulting in the incorrect assignment of specimen BMNH 95.6.7.1 to the slides and as the holotype of *Placospongia carinata*. This specimen BMNH 95.6.7.1, furthermore, has two labels attached to it: one with “*Geodia carinata*”, and one with “*Placospongia melobesioides*”. According to Bowerbank (1874) three specimens had been reviewed for his manuscript: one received from his friend Mr. Thos. Ingall in 1854, one placed by Dr. Baird from the coral to the sponge collection in the BMNH, and one specimen purchased by Bowerbank in 1864. The first mentioned specimen is presumably the holotype, but as this specimen has not been located, I propose to designate the slides R1228-86g-Bk.1390 and R1275-PE01-Bk1390 as representing the holotype of *Placospongia carinata*.

#### Placospongia melobesioides

Gray, 1867

http://species-id.net/wiki/Placospongia_melobesioides

Figure 6

Placospongia melobesioides Gray (1867): figs 1–4.

##### Material examined.

**Holotype**. BMNH 52.4.1.14, Indonesia, Borneo island.

**Vosmaer & Vernhout (1902), Siboga expedition**: RMNH POR. 756, RMNH POR. 761, RMNH POR. 758, RMNH POR. 757, RMNH POR. 760, RMNH POR. 759. **Other material:**: RMNH POR. 4497, RMNH POR. 4496, RMNH POR. 4495, RMNH POR. 4114, RMNH POR. 3978, RMNH POR. 3977, RMNH POR. 3976, RMNH POR. 3942, RMNH POR. 3941, RMNH POR. 3940, RMNH POR. 3939, RMNH POR. 3938, RMNH POR. 3937, RMNH POR. 3935, RMNH POR. 3934, RMNH POR. 3933, RMNH POR. 3932, RMNH POR. 3177, RMNH POR. 3166, RMNH POR. 3154, RMNH POR. 2464, RMNH POR. 2463, ZMA Por. 13097, ZMA Por. 10459 (See Table 3 for full details per specimen)

**Table 3.** Location details of reviewed specimens of *Placospongia melobesioides*.<br/>

**registration number**	**fieldcode**	**country**	**province**	**region**	**island**	**locality**	**habitat**	**latitude**	**longitude**	**depth (m.)**	**date**	**collector**
RMNH POR. 761	#1033	Indonesia		S of Moluccas			sand & rock	04°12'S, 129°20.4'E		45	1899	Siboga expedition
RMNH POR. 756	#660	Indonesia	Nusa Tenggara	N of Sumbawa			sand & rock	07°12.6'S, 118°7.7'E		36	14.ii.1900	Siboga expedition
RMNH POR. 757	#1849	Indonesia	Moluccas	SE of Misool	Banda islands		sand & rock			32	1899	Siboga expedition
RMNH POR. 758	#1847	Indonesia	Moluccas	SE of Misool	Banda islands		sand & rock			32	1899	Siboga expedition
RMNH POR. 759	#1853	Indonesia	Moluccas	SE of Misool	Banda islands		sand & rock			32	1899	Siboga expedition
RMNH POR. 760	#1851	Indonesia	Moluccas	SE of Misool	Banda islands		sand & rock			32	1899	Siboga expedition
RMNH POR. 2463	#Sin05/270306/025	Singapore			Semaku	Pulau Semakau NW side	reef	01°13'70"N, 103°45'61"E		10-12	iii.2006	N.J. de Voogd
RMNH POR. 2464	#Sin05/270306/026	Singapore			Semaku	Pulau Semakau NW side	reef	01°13'70"N, 103°45'61"E		10-12	iii.2006	N.J. de Voogd
RMNH POR. 3154	#LEMD05/30	Indonesia	North Sulawesi		Bunaken	Pangalisang	reef	01°37'26"N, 124°46'55"E		9	24.ix.2006	L.E.Becking
RMNH POR. 3166	#LEMD13/69	Indonesia	North Sulawesi		Bunaken	Pangalisang	reef	01°37'26"N, 124°46'55"E		19	28.ix.2006	L.E.Becking
RMNH POR. 3177	#LEMD22/87	Indonesia	North Sulawesi		Bunaken	Likuan2	reef	01°35'78"N, 124°46'06"E		21	13.x.2006	L.E.Becking
RMNH POR. 3932	#KKB/mol866	Indonesia	East Kalimantan	Berau	Kakaban	Kakaban lake	marine lake	02°08'57.3"N, 118°31'26.4"E		0-2	ix.2008	L.E.Becking
RMNH POR. 3933	#KKB/mol766	Indonesia	East Kalimantan	Berau	Kakaban	Kakaban lake	marine lake	02°08'57.3"N, 118°31'26.4"E		0-2	ix.2008	L.E.Becking
RMNH POR. 3934	#KKB/mol767	Indonesia	East Kalimantan	Berau	Kakaban	Kakaban lake	marine lake	02°08'57.3"N, 118°31'26.4"E		0-2	ix.2008	L.E.Becking
RMNH POR. 3935	#BER113/mol689	Indonesia	East Kalimantan	Berau	Maratua	NE Maratua	reef	02°17'32.3"N, 118°35'26.1"E		5-10	15.viii.2008	N.J. de Voogd
RMNH POR. 3937	#BER107/mol604	Indonesia	East Kalimantan	Berau	Sangalaki	E Sangalaki	reef	02°05'36.6"N, 118°24'15.2"E		5-10	15.viii.2008	L.E.Becking
RMNH POR. 3938	#BER107/mol608	Indonesia	East Kalimantan	Berau	Sangalaki	E Sangalaki	reef	02°05'36.6"N, 118°24'15.2"E		5-10	15.viii.2008	L.E.Becking
RMNH POR. 3939	#BER108/mol601	Indonesia	East Kalimantan	Berau	Sangalaki	W Sangalaki	reef	02°05'07.7"N, 118°23'28.0"E		5-10	15.viii.2008	L.E.Becking
RMNH POR. 3940	#P-YAP1	Micronesia	Yap		Yap		reefflat in front of mangrove	09°31'36.7"N, 138°07'48.7"E		1-3	28.viii.2010	L.E.Becking
RMNH POR. 3941	#P-YAP2	Micronesia	Yap		Yap		reefflat in front of mangrove	09°31'36.7"N, 138°07'48.7"E		1-3	28.viii.2010	L.E.Becking
RMNH POR. 3942	#P-YAP3	Micronesia	Yap		Yap		reefflat in front of mangrove	09°31'36.7"N, 138°07'48.7"E		1-3	28.viii.2010	L.E.Becking
RMNH POR. 3976	#PM-TER02	Indonesia	Moluccas		Ternate		reef			5-10	xi.2009	N.J. de Voogd
RMNH POR. 3977	#PM-TER08	Indonesia	Moluccas		Ternate		reef			5-10	xi.2009	N.J. de Voogd
RMNH POR. 3978	#PM-TER12	Indonesia	Moluccas		Ternate		reef			5-10	xi.2009	N.J. de Voogd
RMNH POR. 4114	#KKB/mol795	Indonesia	East Kalimantan	Berau	Kakaban	Kakaban lake	marine lake	02°08'57.3"N, 118°31'26.4"E		0-2	ix.2008	L.E.Becking
RMNH POR. 4495	#KKB/mol1075	Indonesia	East Kalimantan	Berau	Kakaban	Kakaban lake	marine lake	02°08'57.3"N, 118°31'26.4"E		0-2	ix.2008	L.E.Becking
RMNH POR. 4496	#KKB/mol776	Indonesia	East Kalimantan	Berau	Kakaban	Kakaban lake	marine lake	02°08'57.3"N, 118°31'26.4"E		0-2	ix.2008	L.E.Becking
RMNH POR. 4497	#BER107/mol603	Indonesia	East Kalimantan	Berau	Sangalaki	E Sangalaki	reef	02°05'36.6"N, 118°24'15.2"E		5-10	15.viii.2008	L.E.Becking
ZMA Por. 10459		Seychelles	Mahé		Mahé	NE coast, North East Point	reef	04°34'59.9"S, 055°28'0.1"E		5	8.xii.1992	R.W.M. van Soest
ZMA Por. 10496		Seychelles	Mahé		Mahé	North East Point	reef	04°34'59.9"S, 055°28'0.1"E			14.xii.1992	R.W.M. van Soest
ZMA Por. 13097		Indonesia	South Sulawesi	Spermonde archipelago	Samalona		reef			5-30	27.iv.1997	N.J. de Voogd

##### Description.

Holotype BMNH 52.4.1.14 dry, chalky white angular branches, hard. Other examined material encrusting to branching, hard, thicker specimens slightly compressible. External morphology follows the description of the genus. Size ranging between 5-50 cm, though encrusting specimens may be larger growing within crevices. Ectosome color in life ranging from purple, dark black brown, chocolate brown, orange brown to light beige (Fig. 1, 2). Choanosome pale beige. After preservation color of ectosome is similar to live color.

**Figure 6.**
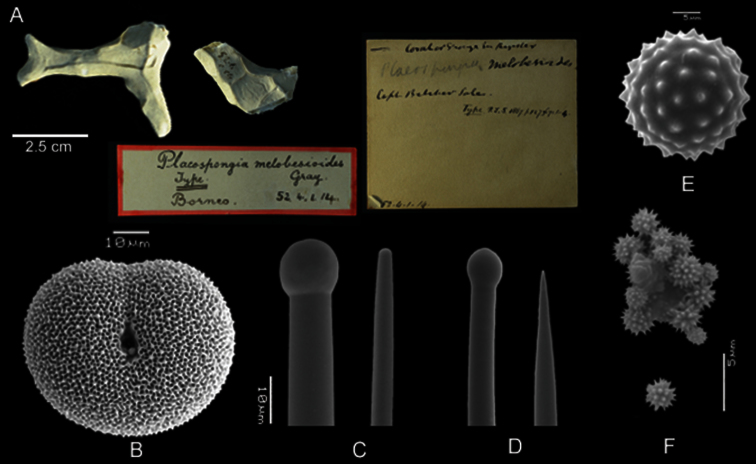
*Placospongia melobesioides* holotype (BMNH 52.4.1.14). **A** Holotype with two labels **B** selenaster **C** large tylostyle (head and blunt end) **D** small tylostyle (head and hastate end) **E** spheraster **F** spherules.

**Spicules.** Holotype BMNH 52.4.1.14 (Fig. 6): Megascleres large straight tylostyles with blunt ends 670-880-1010 × 10-13-18 × 10-16-20 μm, small concave to straight tylostyles with sharp ends 205-293-420 × 5-10-13 × 5-10-13 μm. Microscleres selenasters 58-63-68 × 45-52-68 μm, spherasters 15-17-18 μm (five measurements, not abundant), spherules 1-2-3 *μ* m. The range within the examined material (Table 1): large tylostyles 460-1040 × 5-16 × 8-18 *μ* m, small tylostyles 190-470 × 3-13 × 3-15 *μ* m, selenasters 45-83 × 30-65 *μ* m, spherules 1-3 *μ* m, spherasters only found in singles in some individuals 15-20 *μ* m. Streptasters and microrhabds absent.

**Skeleton.** As description of genus with addition of sporadic spherasters lodged amidst selenasters in cortex and high abundance of spherules in choanosome and cortex.

##### Ecology.

Depth: 0-45m. Reefs, rocky shores, reefflats, mangroves, and marine lakes.

##### Distribution.

Type locality: Borneo. Distribution from Seychelles to Micronesia (Fig. 9, Table 3). Possibly further east to Central Pacific.

##### Remarks.

In the original description by Gray (1867) there is no mention of two size classes of tylostyles. I reexamined the original slide and conclude that the holotype does contain two size classes of tylostyles. The Systema Porifera indicates that the holotype has two size classes, the large 720-963-1200 × 13-14.1-19 μm and the small 350-438.8-560 × 8-9.1-10.5 μm, based on 10 measurements per spicule type (Rützler 2002). These measurements deviate from the holotype measurements in the present study that were based on 25 measurements per spicule type (670-880-1010 × 10-13-18 μm and 205-293-420 × 5-10-13 μm respectively), and also deviate from the range of sizes within the examined material of this study (Table 1). There is great variation in tylostyle length and spherasters are only sporadically present, often absent.

#### Placospongia mixta

Thiele 1900

http://species-id.net/wiki/Placospongia_mixta

Figure 7

Placospongia mixta Thiele, 1900: Plate III, fig. 25.

##### Material examined.

**Holotype**. ZMB 3204, Indonesia, Moluccas, Ternate.

**Vosmaerand & Vernhout (1902), Siboga expedition**: RMNH POR. 753, RMNH POR. 751, RMNH POR. 745, RMNH POR. 742. **Other material:** RMNH POR. 4494, RMNH POR. 4493, RMNH, POR. 4492, RMNH POR. 4491, RMNH POR. 4490, RMNH POR. 4489, RMNH POR. 4113, RMNH POR. 4112, RMNH, POR. 3979, RMNH POR. 3975, RMNH POR. 3974, RMNH POR. 3973, RMNH POR. 3972, RMNH POR. 3971, RMNH POR. 3970, RMNH POR. 3969, RMNH POR. 3968, RMNH POR. 3967, RMNH POR. 3966, RMNH POR. 3965, RMNH POR. 3964, RMNH POR. 3963, RMNH POR. 3962, RMNH POR. 3961, RMNH POR. 3960, RMNH POR. 3959, RMNH, POR. 3163, RMNH POR. 3158, RMNH POR. 3157, RMNH POR. 3155, RMNH POR. 3148, ZMA Por. 10495, ZMA Por. 896 (See Table 4 for full details per specimen)

**Table 4.** Location details of reviewed specimens of *Placospongia mixta*.<br/>

**registration number**	**fieldcode**	**country**	**province**	**region**	**island**	**locality**	**habitat**	**latitude**	**longitude**	**depth**	**date**	**collector**
RMNH POR. 753	#311	Indonesia	West Papua	E. of Misool			sand & rock	01°42.5'S, 130°47.5'E		32	20.viii.1899	Siboga expedition
RMNH POR. 751	#1857	Indonesia	West Papua	E. of Misool			sand & rock	01°42.5'S, 130°47.5'E		32	20.viii.1899	Siboga expedition
RMNH POR. 745	#577	Indonesia	South Sulawesi		N. of Kabia	Saleyer anchorage	sand & rock			36	20.viii.1899	Siboga expedition
RMNH POR. 742	#163a	Indonesia	Moluccas		Aru	Pearl Banks, anchorage off Pulu Jedan	reef			13	23.xii.1899	Siboga expedition
RMNH POR. 3148	#LEMD04/21	Indonesia	North Sulawesi		Bunaken	Likuan 2	reef	01°35'78"N, 124°46'06"E		15	24.ix.2006	L.E. Becking
RMNH POR. 3155	#LEMD06/32	Indonesia	North Sulawesi	Lembeh Strait		Nudi Reed Reed	reef	01°24'06"N, 125°12'22"E		21	25.ix.2006	L.E. Becking
RMNH POR. 3157	#LEMD08/39	Indonesia	North Sulawesi	Lembeh Strait		Nudi Fols	reef	01°27'26"N, 125°13'05"E		6	25.ix.2006	L.E. Becking
RMNH POR. 3158	#LEMD08/42	Indonesia	North Sulawesi	Lembeh Strait		Nudi Fols	reef	01°27'26"N, 125°13'05"E		8	25.ix.2006	L.E. Becking
RMNH POR. 3163	#LEMD11/52	Indonesia	North Sulawesi		Bunaken	0.5-1km W. of Park administration office	reef	01°36'57"N, 124°45'41"E		8	27.ix.2006	L.E. Becking
RMNH POR. 3959	#KKB/mol827	Indonesia	East Kalimantan	Berau	Kakaban	Kakaban lake	marine lake	02°08'57.3"N, 118°31'26.4"E		0-2	ix.2008	L.E.Becking
RMNH POR. 3960	#KKB/mol829	Indonesia	East Kalimantan	Berau	Kakaban	Kakaban lake	marine lake	02°08'57.3"N, 118°31'26.4"E		0-2	ix.2008	L.E.Becking
RMNH POR. 3961	#KKB/mol851	Indonesia	East Kalimantan	Berau	Kakaban	Kakaban lake	marine lake	02°08'57.3"N, 118°31'26.4"E		0-2	ix.2008	L.E.Becking
RMNH POR. 3962	#BER111/mol1203	Indonesia	East Kalimantan	Berau	Kakaban	SW Kakaban	reef	02°08'07.5"N, 118°30'23.3"E		10	17.viii.2008	N.J. de Voogd
RMNH POR. 3963	#BER111/1209	Indonesia	East Kalimantan	Berau	Kakaban	SW Kakaban	reef	02°08'07.5"N, 118°30'23.3"E		10	17.viii.2008	N.J. de Voogd
RMNH POR. 3964	#BER111/1213	Indonesia	East Kalimantan	Berau	Kakaban	SW Kakaban	reef	02°08'07.5"N, 118°30'23.3"E		10	17.viii.2008	N.J. de Voogd
RMNH POR. 3965	#BER111/mol1219	Indonesia	East Kalimantan	Berau	Kakaban	SW Kakaban	reef	02°08'07.5"N, 118°30'23.3"E		10	17.viii.2008	N.J. de Voogd
RMNH POR. 3966	#RAJ23/mol195	Indonesia	West Papua	Raja Ampat	Gam	Ctenophore lake	marine lake	00°27'17.5"S, 130°29'33.8"E		0-2	xi.2007	L.E. Becking
RMNH POR. 3967	#RAJ23/mol187	Indonesia	West Papua	Raja Ampat	Gam	Ctenophore lake	marine lake	00°27'17.5"S, 130°29'33.8"E		0-2	xi.2007	L.E. Becking
RMNH POR. 3968	#RAJ64/mol429	Indonesia	West Papua	Raja Ampat	Waigeo	Teluk Mayabilit	reef	00°18'17.0"S, 130°54'15.6"E		10	xii.2007	L.E. Becking
RMNH POR. 3969	#RAJ64/mol430	Indonesia	West Papua	Raja Ampat	Waigeo	Teluk Mayabilit	reef	00°18'17.0"S, 130°54'15.6"E		10	xii.2007	L.E. Becking
RMNH POR. 3970	#RAJ64/mol431	Indonesia	West Papua	Raja Ampat	Waigeo	Teluk Mayabilit	reef	00°18'17.0"S, 130°54'15.6"E		10	xii.2007	L.E. Becking
RMNH POR. 3971	#RAJ64/mol432	Indonesia	West Papua	Raja Ampat	Waigeo	Teluk Mayabilit	reef	00°18'17.0"S, 130°54'15.6"E		10	xii.2007	L.E. Becking
RMNH POR. 3972	#RAJ64/mol433	Indonesia	West Papua	Raja Ampat	Waigeo	Teluk Mayabilit	reef	00°18'17.0"S, 130°54'15.6"E		10	xii.2007	L.E. Becking
RMNH POR. 3973	#RAJ39/mol249	Indonesia	West Papua	Raja Ampat	Fam		rocky shore	00°36'01.5"S, 130°45'08"E		0-1	xi.2007	L.E. Becking
RMNH POR. 3974	#RAJ39/mol250	Indonesia	West Papua	Raja Ampat	Fam		rocky shore	00°36'01.5"S, 130°45'08"E		0-1	xi.2007	L.E. Becking
RMNH POR. 3975	#RAJ39/mol254	Indonesia	West Papua	Raja Ampat	Fam		rocky shore	00°36'01.5"S, 130°45'08"E		0-1	xi.2007	L.E. Becking
RMNH POR. 3979	#KKB/mol 779	Indonesia	East Kalimantan	Berau	Kakaban	Kakaban lake	marine lake	02°08'57.3"N, 118°31'26.4"E		0-2	ix.2008	L.E.Becking
RMNH POR. 4112	#P-TER11	Indonesia	Moluccas		Ternate		reef				xi.2009	N.J. de Voogd
RMNH POR. 4113	#P-TER22	Indonesia	Moluccas		Ternate		reef				xi.2009	N.J. de Voogd
RMNH POR. 4489	#KKB/mol721	Indonesia	East Kalimantan	Berau	Kakaban	Kakaban lake	marine lake	02°08'57.3"N, 118°31'26.4"E		0-2	ix.2008	L.E.Becking
RMNH POR. 4490	#KKB/mol830	Indonesia	East Kalimantan	Berau	Kakaban	Kakaban lake	marine lake	02°08'57.3"N, 118°31'26.4"E		0-2	ix.2008	L.E.Becking
RMNH POR. 4491	#BER109/mol629	Indonesia	East Kalimantan	Berau		lighthouse near Berau river	reef	02°09'49.9"N, 118°10'12.8"E		10	16.viii.2008	L.E.Becking
RMNH POR. 4492	#BER111/mol666	Indonesia	East Kalimantan	Berau	Kakaban	SW Kakaban	reef	02°08'07.5"N, 118°30'23.3"E		10	17.viii.2008	N.J. de Voogd
RMNH POR. 4493	#RAJ64/mol428)	Indonesia	West Papua	Raja Ampat	Waigeo	Teluk Mayabilit	reef	00°18'17.0"S, 130°54'15.6"E		10	xii.2007	L.E. Becking
RMNH POR. 4494	#RAJ23/mol199	Indonesia	West Papua	Raja Ampat	Gam	Ctenophore lake	marine lake	00°27'17.5"S, 130°29'33.8"E		0-2	xi.2007	L.E. Becking
ZMA Por. 896		Indonesia	South Sulawesi		SW Salayer	reef N of Pulau Bahuluang	reef	06°27'00"S, 120°25'48"E		10-45	30.ix.1984	R.W.M. van Soest (Snellius Expedition II)
ZMA Por. 10495		Seychelles		Mahé	Mahé	SE coast near Pointe Cocos		04°45'00"S, 055°32'60"E		35-45	24.xii.1992	R.W.M. van Soest

##### Description.

Holotype ZMB 3204 encrusting, size 5 × 2.5 cm and thickness 1–5 mm (as described by Thiele, now very small fragment), white after preservation in alcohol. The majority of the reviewed material is encrusting with a thickness of 4-10mm, but branching specimens also occur. External morphology follows the description of the genus. Color of the ectosome can be red, orange, brown orange, dark brown, chocolate brown, milk coffee brown, cream, or white (Fig. 1, 2). Color of choanosome is pale beige. After preservation in ethanol color is similar to live specimens, but lighter shade.

**Spicules.** Holotype ZMB 3204 (Fig. 6) Megascleres large straight tylostyles with blunt/rounded point 355-672-940 × 7.5-12-17.5 × 7.5-16-20 μm, small straight tylostyles with sharp point 165-226-275 × 2.5-6-7.5 × 2.5-8-10 μm; microscleres selenasters 55-70-75 × 42.5-55-72.5 μm, spherasters (abundant) 20-25-30 μm, streptasters typically with well developed axis and with 4-9 rays with hastate tips, rays are smooth or can be spined, but do not have bifurcations of the tips 15-24-32.5 × 2.5-8-12.5 μm; acanthose microrhabs with straight or zig-zag axis 5-7-10 × <2.5 *μ* m.The range within the examined material (Table 1): large tylostyles 460-1250 × 8-23 × 10-25 *μ* m, small tylostyles 120-430 × 3-15 × 2-15 *μ* m, selenasters 50-85 × 22-73 *μ* m, spherasters 13-30 *μ* m, streptasters 15-35 × 2-15 *μ* m, rays 5-18 × 1-2.5 *μ* m.

**Skeleton.** As description of genus with addition that microrhabds form a layer over and amidst the selenaster cortex and are also prevalent in choanosomal tissue. Streptasters scattered in choanosome. Spherasters amidst selenasters in cortex and scattered in choanosome.

**Figure 7.**
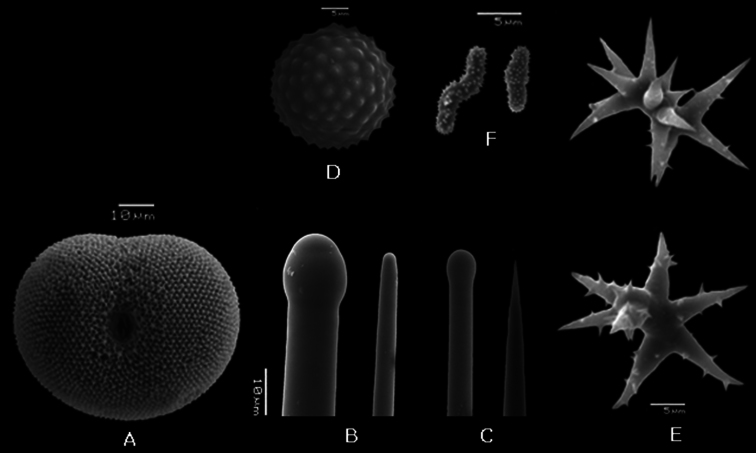
*Placospongia mixta* holotype (ZMB 3204). **A** selenaster **B** large tylostyle (head and blunt end) **C** small tylostyle (head and hastate end) **D** spheraster **E** streptasters **F** microacanthose microrhabds.

##### Distribution.

East African coast to eastern Indonesia (Fig. 9, Table 4). Possibly further east to Central Pacific. Pulitzer-Finali (1993) identified a ‘*P. carinata’* from East Africa (Mombasa) that fits the description of *Placospongia mixta* based on the length of the tylostyles (up to 1200 *μ* m) and the presence of spherasters, but no *Placospongia mixta* specimens were observed in the Seychelles material deposited at ZMA.

##### Ecology.

Depth 0–45m. Common in reefs, also occurs in marine lakes.

##### Remarks.

In 1900 Thiele described a new species named *Placospongia mixta*, which was originally identified as *Placospongia melobesioides* by Kieschnick (1896). The specific epithet *mixta* was given because the specimen contained a mixture of spicules: both spirasters like *Placospongia carinata* as well as large spherasters like *Placospongia intermedia* and *Placospongia melobesioides*, which are absent in *Placospongia carinata*. In 1902 Vosmaer & Vernhout decided that *Placospongia mixta* was a junior synonym of *Placospongia carinata*, because they saw no distinction between the different shapes of streptasters and stated that spherasters are never very abundant – in some ‘exceedingly rare and in some we failed to find them at all’ – and could therefore not be seen as a distinguishing character. The specimens that were studied by Vosmaer and Vernhout (1902) were collected in Indonesia during the Siboga Expedition (1899-1900) and are housed in the collection of the Naturalis Biodiversity Center (Leiden, The Netherlands). In the present study these specimens were reexamined. After inspection, the specimens labeled ‘*Placospongia carinata*’ could be clearly and consistently divided into two species: *Placospongia carinata* without spherasters, with streptasters displaying bifurcating tips, and tylostyles up to 980 *μ* m, and *Placospongia mixta* with abundant spherasters, with streptasters displaying hastate tips, and tylostyles up to 1250 *μ* m. In none of the specimens of Vosmaer and Vernhout (1902), nor of the other specimens reviewed for this study was there a mixture of the two types of streptasters. These two species also show molecular distinction in both mitochondrial and nuclear markers (Fig. 10, 11, Table 6, 7).

#### Placospongia santodomingoae sp. n.

urn:lsid:zoobank.org:act:3C4F2599-15C0-4075-BD3B-8C6439C8F821

http://species-id.net/wiki/Placospongia_santodomingoae

Figure 8

##### Holotype.

RMNH POR. 4486, Indonesia, East Kalimantan province, Maratua island, Buli Halo anchialine pool, 02°11'16.4"N, 118°37'06.4"E, 0–1m. depth, xi.2008, coll. N.K. Santodomingo & Estradivari, #BER128/mol1147. **Paratypes.** RMNH POR. 4487, Indonesia, East Kalimantan province, Maratua island, Buli Halo anchialine pool, 02°11'16.4"N, 118°37'06.4"E, 0–1m. depth, xi.2008, coll. N. K. Santodomingo & Estradivari; RMNH POR. 4488, Indonesia, East Kalimantan province, Maratua island, Buli Halo anchialine pool, 02°11'16.4"N, 118°37'06.4"E, 0–1m. depth, xi.2008, coll. N. K. Santodomingo & Estradivari, #BER128/1156.

##### Description.

**Figure 8.**
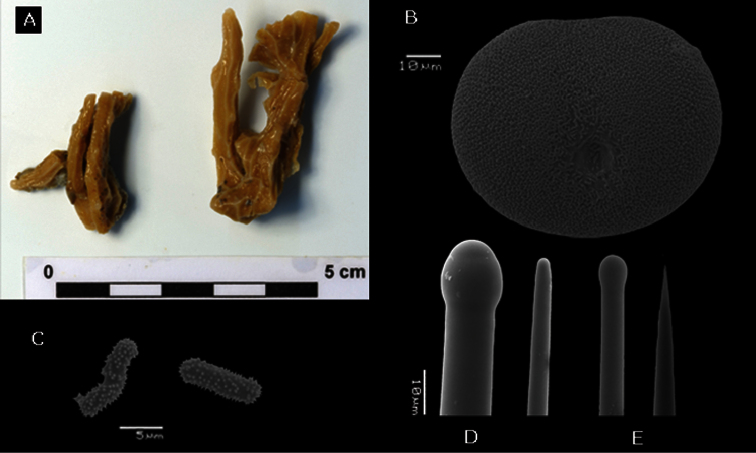
*Placospongia santodomingoae* sp. n. (RMNH POR. 4486). **A** ethanol preserved specimen **B** selenaster **C** large tylostyle (head and blunt end) **D** small tylostyle (head and hastate end) **E** microrhabds.

Holotype and paratypes are branching and encrusting, size 8cm in length. Total size of specimens *in situ* is hard to establish as parts of the body may be encrusting within cracks. Alcohol preserved and live specimens are hard but slightly compressible. The surface is made up with typical *Placospongia* cortical plates separated by contractible grooves which form a network on the surface. Oscules are present in the grooves. Live color of holotype was dark brown, the paratypes were orange, and these colors were mostly retained after alcohol preservation (Fig. 8A).

**Spicules.** Holotype (Fig. 8) megascleres large straight tylostyles with blunt point 430-605.5-660 × 13-15.5-20 × 13-18.1-23 μm, small straight tylostyles with sharp point 240-261.3-290 × 5-7.2-8 × 5-8.8-10 μm; microscleres selenasters 80-84.8-90 × 60-67.3-75 μm, acanthose microrhabds 8-12.3-18 × 2.5-2.7-3.5 μm. Range of the paratypes (Table 1) large straight tylostyles with blunt point 430-760 × 13-20 × 15-23 μm, small straight tylostyles with sharp point 190-380 × 5-13 × 8-15 μm, microscleres selenasters 63-93 × 58-75 *μ* m, acanthose microrhabds 5-20 × 2.5-3.5 *μ* m.

**Skeleton.** The cortical plates consist of densely packed selenasters, microrhabds form a layer over and amidst this selenaster cortex and are also prevalent in choanosomal tissue. Developmental stages of selenasters occur throughout the choanosome. Tylostyle tracts support the margins of the cortical plates in radial tracts from the centre core (consisting of densely packed selenaster) to the cortical plates. The sharp ends of the smaller tylostyles can be projected beyond the cortex surface.

##### Distribution.

**Figure 9.**
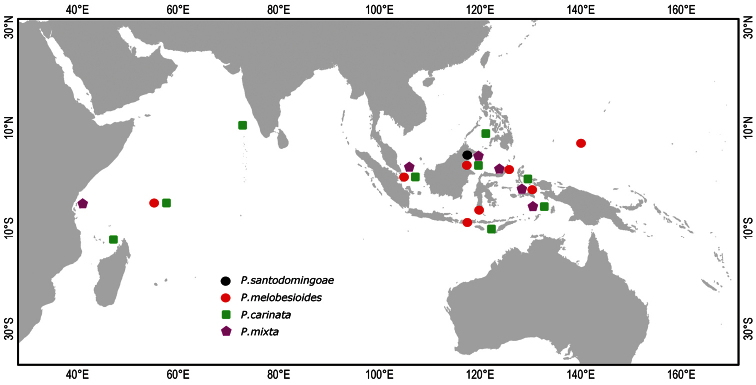
Distribution of *Placospongia* spp. in the Indo-West Pacific. Location of symbols is approximate.

Presently only recorded from Buli Halo anchialine pool on Maratua island, Berau, East Kalimantan, Indonesia (Fig. 9). For a full description of the pool, see Becking et al. (2011)

##### Ecology.

Depth 0–2m. occurs in anchialine pool, can be exposed to air during low tide and can tolerate great fluctuations in salinity (from 24 to 33 ‰).

##### Etymology.

Named in honor of Nadiezhda K. Santodomingo, the collector of the types, for her years of tireless work in marine science including anchialine research.

##### Remarks.

*Placospongia santodomingoae* sp. n. is similar to *Placospongia carinata*,yet lacks streptasters and has shorter tylostyles. *Placospongia santodomingoae* sp. n. likewise differs from *Placospongia mixta* by the absence of streptasters as well as the absence of spherasters. *Placospongia santodomingoae* sp. n. differs from *Placospongia anthosigma* by the absence of anthosigma, and by having hastate endings of the smaller tylostyles.

#### Geodia labyrinthica

(Kirkpatrick, 1903)

http://species-id.net/wiki/Geodia_labyrinthica

Placospongia labyrinthica Kirkpatrick 1903: Plate V fig. 1a–b, Plate VI fig. 1a–f.

##### Reviewed material.

**Holotype.** BMNH 02.11.16.1, South Africa, East London Coast, 33°06'30"S, 028°11'E.

**Spicules.** Megascleres styles, oxea; microscleres sterrasters, chiasters

##### Remarks.

This species was originally described as ‘*Placospongia labyrinthica’*,butdoes not have the characteristic cortical plates of *Placospongia*. The specimen furthermore has sieve pores, sterrasters with star-like plates, euasters, styles and oxea characteristic of the Geodiidae. In the original description, Kirkpatrick (1903) stated “the presence of chiasters is so exceptional that I thought at first that I had to deal with a geodine sponges, but there were no triaenes to be found” and as a result placed this species in the *Placospongia* rather than *Geodia*. Genus transfer to *Geodia* is, however, required as suggested on the World Porifera Database (van Soest et al. 2011).

### Identification key for Indo-Pacific species of *Placospongia*

**Table:** 

1	Streptasters absent	2
–	Streptasters present	3
2	Spherules present	*Placospongia melobesioides*
–	Spherules absent	4
3	Streptasters have rays with birfurcating ends	*Placospongia carinata*
–	Streptasters have rays with hastate ends, spherasters present	*Placospongia mixta*
4	Spherasters present, microrhabds with short spines spirally places on shaft	*Placospongia anthosigma*
–	Spherasters absent, acanthose microrhabds present	*Placospongia santodomingoae* sp.n.

## Genetic data analysis

**Figure 10.**
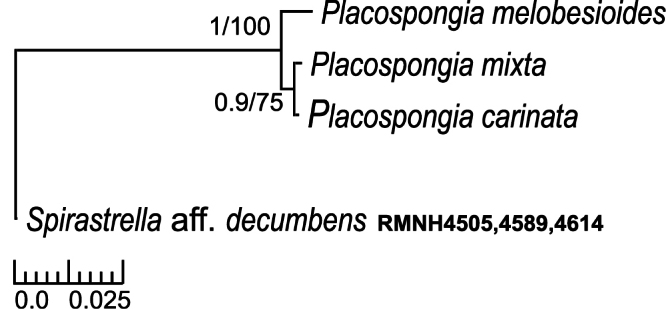
Bayesian/maximum likelihood phylograms of Cytochrome Oxidase I (COI) sequences from Indo-Pacific *Placospongia* spp. See Table 5 for GenBank accession numbers. Only posterior probabilities of >90 and maximum likelihood values of >70 indicated. Scale bar indicates substitutions/site.

All sequences were submitted to GenBank with accession numbers KC848421 - 41 (Table 5). Final alignments (excluding primers) were obtained for the sponge *Placospongia* of 581 bp for COI with three genetic variants (28 individuals) and 13 polymorphic sites. The three genetic variants correspond to the three species *Placospongia melobesioides*, *Placospongia mixta*, and *Placospongia carinata* that represent monophyletic groups which are strongly supported by both Bayesian and maximum likelihood inference methods (Fig. 10). There was no intra-specific variation within each species, regardless of geographic locality. The inter-specific *p-* distances ranged between 0.5-2.1% (Table 6).There were 11 substitutions between *Placospongia melobesioides* and *Placospongia carinata*, 12 substitutionsbetween *Placospongia melobesioides* and *Placospongia mixta*, and three substitutions between *Placospongia mixta* and *Placospongia carinata*. The specimens of *Placospongia carinata* and of *Placospongia santodomingoae* sp. n. had identical genotypes for COI. No molecular work could be done on the dried holotype of *Placospongia anthosigma* and fresh material was not available.

**Figure 11.**
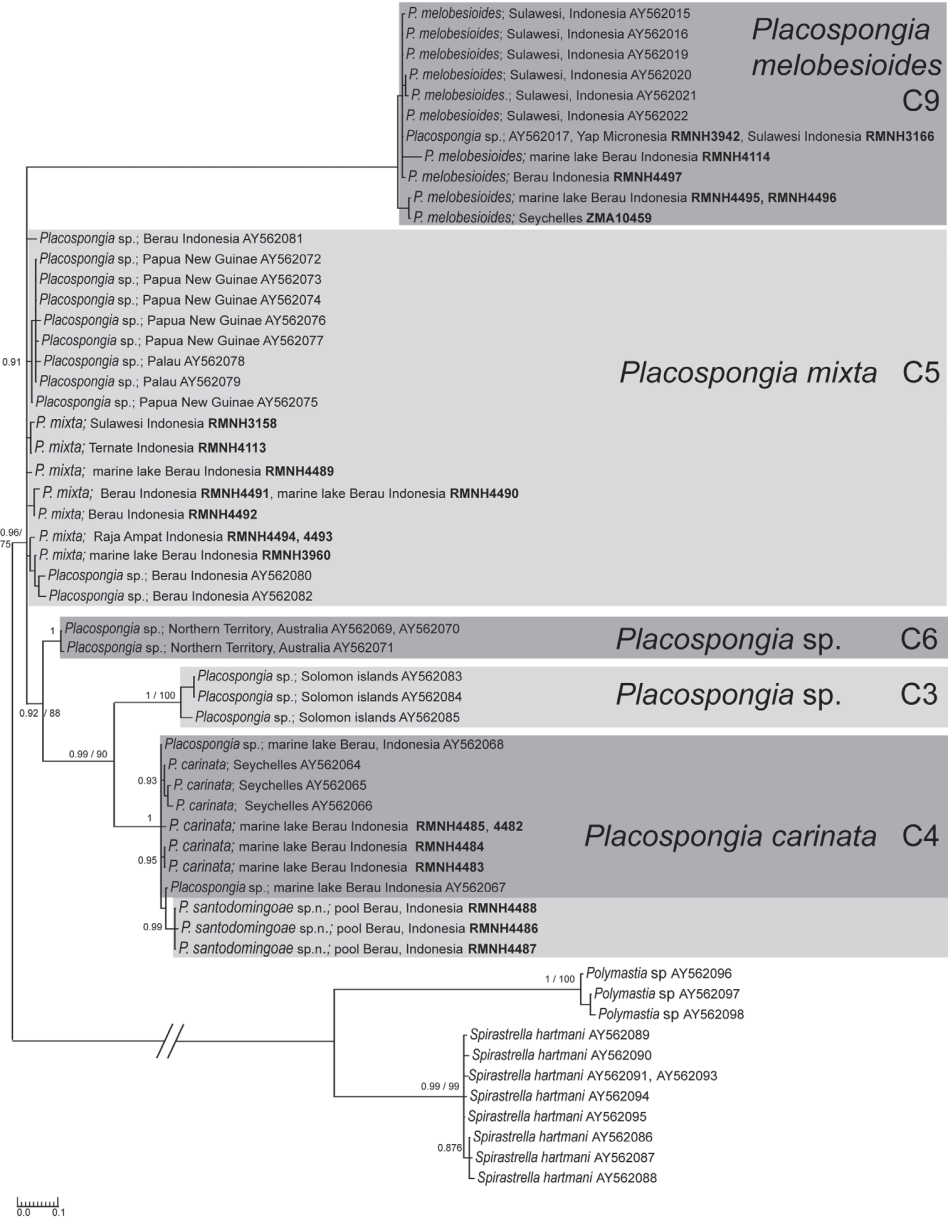
Bayesian/maximum likelihood phylograms of genotypes of the internal transcribed spacer region of nuclear ribosomal operons (ITS) of Indo-Pacific *Placospongia* spp. found in this study and related species from the same genus collected from GenBank. Clades C3, C4, C5, C6 & C9 refer to the clades presented in the study by Nichols and Barnes (2005). Taxon labels are organized as follows: Specimen - Locality - Genbank code or RMNH POR. Number. Only posterior probabilities of >90 and maximum likelihood values of >70 indicated. Scale bar indicate substitutions/site.

Final alignments (excluding primers) of 720 bp were obtained for ITS with 18 genetic variants from the present study (22 individuals). An additional 27 genetic variants from GenBank (for GenBank accession numbers see Fig. 11) were included in the phylogenetic analysis. The ITS sequences represented five clades that were strongly supported by both Bayesian and maximum likelihood inference methods (Fig. 11). These five divergent clades (see Table 7 for uncorrected *p-* distances) correspond to the clades C3, C4, C5, C6, and C9 as presented by the study of Nichols and Barnes (2005). Clade C9 represents specimens of the species *Placospongia melobesioides*, clade C5 *Placospongia mixta*, and clade C4 *Placospongia carinata*. Clades C6 is represented by one specimen from the Solomon Islands (QM317896) and clade C3 by one specimen from Bynoe Harbour, Northern Territory, Australia (QM303439); none of the samples that were sequenced in the present study fell into either C3 or C6 clade. The specimens of *Placospongia santodomingoae* sp. n. represented a separate lineage within the *Placospongia carinata* clade (C4) which was supported by Bayesian inference, but not by maximum likelihood analysis. The *p-* distancebetween *Placospongia carinata* specimens and the specimens of *Placospongia santodomingoae* sp. n. was 0.6%.

**Table 5.** Specimens of *Placospongia* studied for DNA analysis. Genbank accession numbers provided for sequences of Cytochrome Oxidase I (COI) and internal transcribed spacer region (ITS).<br/>

**Registration number**	**Species**	**COI**	**ITS**
RMNH POR. 4482	*Placospongia carinata*	KC848441	KC848429
RMNH POR. 4483	*Placospongia carinata*	KC848441	KC848427
RMNH POR. 4484	*Placospongia carinata*	KC848441	KC848428
RMNH POR. 4485	*Placospongia carinata*	KC848441	KC848429
ZMA Por. 10727	*Placospongia carinata*	KC848441	-
ZMA Por. 11367	*Placospongia carinata*	KC848441	-
RMNH POR. 2464	*Placospongia melobesioides*	KC848439	-
RMNH POR. 3942	*Placospongia melobesioides*	KC848439	KC848422
RMNH POR. 3976	*Placospongia melobesioides*	KC848439	-
RMNH POR. 4114	*Placospongia melobesioides*	KC848439	KC848426
RMNH POR. 4495	*Placospongia melobesioides*	KC848439	KC848436
RMNH POR. 4496	*Placospongia melobesioides*	KC848439	KC848436
RMNH POR. 4497	*Placospongia melobesioides*	KC848439	KC848437
RMNH POR.3166	*Placospongia melobesioides*	KC848439	KC848422
ZMA Por. 10459	*Placospongia melobesioides*	KC848439	KC848438
RMNH POR. 3158	*Placospongia mixta*	KC848440	KC848421
RMNH POR. 3960	*Placospongia mixta*	KC848440	KC848423
RMNH POR. 3979	*Placospongia mixta*	KC848440	-
RMNH POR. 4113	*Placospongia mixta*	KC848440	KC848425
RMNH POR. 4489	*Placospongia mixta*	KC848440	-
RMNH POR. 4490	*Placospongia mixta*	KC848440	KC848433
RMNH POR. 4491	*Placospongia mixta*	KC848440	KC848433
RMNH POR. 4492	*Placospongia mixta*	KC848440	KC848434
RMNH POR. 4493	*Placospongia mixta*	KC848440	KC848435
RMNH POR. 4494	*Placospongia mixta*	KC848440	KC848435
RMNH POR. 4486	*Placospongia santodomingoae* sp. n.	KC848441	KC848430
RMNH POR. 4487	*Placospongia santodomingoae* sp. n.	KC848441	KC848431
RMNH POR. 4488	*Placospongia santodomingoae* sp. n.	KC848441	KC848432

**Table 6.** The number of base differences per site from averaging over all Cytochrome Oxidase I (COI) sequence pairs between *Placospongia* spp. groups are shown (uncorrected *p*-distances). Standard error estimate (s) are shown above the diagonal in italic. The analysis involved 30 nucleotide sequences. There was no within-group difference. *Spirastrella* aff. *decumbens* was used as outgroup in the phylogenetic inference (see Fig. 10).<br/>

**%*p*-distance COI**	***Placospongia melobesioides***	***Placospongia mixta***	***Placospongia carinata***	***Spirastrella* aff. *decumbens***
*Placospongia melobesioides*	*	*0.6*	*0.6*	*1.3*
*Placospongia mixta*	2.1	*	*0.3*	*1.2*
*Placospongia carinata*	1.9	0.5	*	*1.3*
*Spirastrella* aff. *decumbens*	12.2	11.5	11.7	*

**Table 7.** The number of base differences per site from averaging over all internal transcribed spacer (ITS) sequence pairs between *Placospongia* spp. groups are shown (uncorrected *p*-distances). Standard error estimate (s) are shown above the diagonal. All positions with less than 5% site coverage were eliminated. Black cursive along the diagonal indicates within-group uncorrected *p-* distance. The analysis involved 73 nucleotide sequences. C9, C5, C6, C4, C3 refer to five clades in the Indo-West Pacific *Placospongia* as presented in Fig. 11.<br/>

**%*p*-distance ITS**	***Placospongia melobesioides***	***Placospongia mixta***	***Placospongia carinata***	***Placospongia santodomingoae* sp. n.**	**C9**	**C5**	**C6**	**C4**	**C3**
*Placospongia melobesioides*	***0.1***	*1.3*	*1.4*	*1.4*	*0.3*	*1.3*	*1.3*	*1.3*	*1.3*
*Placospongia mixta*	13.8	***0.7***	*0.9*	*0.9*	*1.2*	*0.2*	*0.5*	*0.9*	*0.9*
*Placospongia carinata*	14.7	6.3	***0.4***	*0.2*	*1.3*	*0.9*	*0.9*	*0.2*	*0.9*
*Placospongia santodomingoae* sp. n.	13.2	5.8	0.6	***1.6***	*1.3*	*0.9*	*0.9*	*0.3*	*0.9*
C9	0.9	13.5	14.6	13.6	***0.1***	*1.2*	*1.2*	*1.2*	*1.2*
C5	13.5	0.9	6.6	6.1	12.9	***0.7***	*0.5*	*0.8*	*0.9*
C6	14	2.2	6.4	6.1	13.2	2.2	***0.1***	*0.8*	*0.8*
C4	14.8	6.3	0.5	0.9	14.3	6.3	6	***0.4***	*0.8*
C3	15.2	7.1	6.1	5.9	14.5	6.9	6.3	5.6	***0.9***

## Discussion

### Different species

In the Indo-West Pacific at least five species of the genus *Placospongia* can be identified based on spicule morphology: *Placospongia anthosigma*, *Placospongia carinata*, *Placospongia mixta*, *Placospongia melobesioides*, and *Placospongia santodomingoae* sp. n. *Placospongia melobesioides*, *Placospongia carinata*, and *Placospongia mixta* can be distinguished with the DNA barcode marker (COI) and a nuclear marker (ITS). The species *Placospongia santodomingoae* sp. n. and *Placospongia carinata* have the same sequence of COI. The sequence variation of COI in sponges can be low (e.g. Wörheide 2006, Xavier et al. 2010, Pöppe et al. 2011) and this is also the case in species of *Placospongia*, e.g. only 0.5% nucleotide distance between the species *Placospongia mixta* and *Placospongia carinata*.There is furthermore no intraspecific variation in COI within each of the *Placospongia* species, not even between populations at 1000s of km distance from each other (e.g. specimens from the Seychelles are identical with specimens from Indonesia). The phylogenetic inference based on the ITS sequences does show a supported clade of *Placospongia santodomingoae* sp. n. within the clade of *Placospongia carinata* (Fig. 11),though the degree of divergence between the two species is low (0.6%) (Table 7). *Placospongia santodomingoae* sp. n. should, however, be designatedas a new species based on the spicule morphology: the absence of a distinguishing spicule type (streptasters) and consistently shorter and thicker tylostyles (maximum 760 × 20 μm) compared to *Placospongia carinata* (maximum 980 × 17.5 μm) are valid arguments to distinguish a separate species within this genus. The specimens of *Placospongia santodomingoae* sp. n. were collected from an anchialine pool. This kind of isolated environment has previously been shown to contain small, rapidly evolving populations, and many rare species across a large spectrum of taxa (e.g. Holthuis 1973, Tomascik and Mah 1994, Dawson and Hamner 2005, Becking et al. 2011, Becking et al. *in press*). The divergence of *Placospongia santodomingoae* sp. n. from *Placospongia carinata* is likely too recent to be expressed in the molecular markers that were used. Other, faster evolving, molecular markers might show a more distinct separation between species, but for the present significant morphometric differences in spicules are reliable characters in separating these sister species.

A molecular phylogeny using the internal transcribed spacer region (ITS) showed that there were five distinct clades within the genus *Placospongia* in the Indo-West Pacific (clades C3, C4, C5, C6 & C9) (Nichols & Barnes, 2005). Nichols and Barnes (2005) indicated that their results presented a conundrum that “specimens collected from Indonesian marine lakes that have been isolated from the surrounding marine environment since the Pleistocene are undifferentiated from individuals collected from the Seychelles indicating that populations from these geographically disparate regions are, or have recently been, connected by gene flow despite the lack of evidence of connectivity between these lakes and nearby reefs.” It is important to note here that the authors did not investigate the spicule morphology of the specimens in their study, while it is in fact the spicules that can largely explain the presented conundrum. In the present study over 30 specimens from the marine lakes Kakaban and Maratua and the adjacent reefs have been reviewed as well as the specimens from the ZMA Por. collection that were used in the Nichols and Barnes (2005) study. Clade C4 represents the material from the Seychelles (ZMA Por.11367) together with the marine lakes and can all be morphologically identified as *P. carinata sensu stricto*. The samples from the lakes and the Seychelles are thus conspecific, but the populations of the two locations are necessarily connected by gene flow. Subsequently clade C9 is *Placospongia melobesioides* (specimens from Indonesia, Miscronesia and the Seychelles) and clade C5 is of *Placospongia mixta* (specimens from Indonesia, Palau and Papua New Guinae). This explains three of the five clades from the Indo-West Pacific and leaves two undetermined: clade C3 represented by one specimen from Bynoe Harbour, Northern Territory, Australia (QM303439), and clade C6 represented by one specimen from the Solomon Islands (QM317896). The morphology of these specimens should be further studied in order to correctly identify the species and determine if they may represent morphologically cryptic species.

### Natural variation

Each of the five species of the genus *Placospongia* in the Indo-West Pacific can be distinguished based on the composition and morphology of spicules. The external morphology, however, does not allow species distinction. The most common species from the tropical Indo-West Pacific (*Placospongia melobesioides*, *Placospongia mixta*, and *Placospongia carinata*) can have both encrusting and branching growth forms displaying a variety of colors from white to dark brown. The only observed consistent pattern was that all the red specimens belonged to *Placospongia mixta*,while all the dark black-brown specimens belonged to *Placospongia melobesioides*.These two colors may be useful for field identifications, yet both species can also display the range of other colors (white, cream, beige, light brown) as well. The density of canals/ridges (or size of cortical plates) appears to be related to environment as this is higher in specimens from high sediment locations such as the marine lakes than in specimens from the reefs (Fig. 1, 2). Within each species there is also some natural variation in the range of tylostyle length and spicule morphology. The streptaster morphology varies within species and even within individuals. Within one individual the number of rays can vary from 4-10 (Figs 3, 4) and between individuals the decoration and size of spines can be diverse. For example the streptasters of *Placospongia carinata* specimens from Haji Buang marine lake are micro-acanthose while the specimens from other locations are not. Spherasters are always present and abundant in *Placospongia mixta* and *Placospongia anthosigma*,but are in low abundances or absent in *Placospongia melobesioides*, as has been indicated previously by Vosmaer and Vernhout (1902). In *Placospongia carinata* and *Placospongia santodomingoae* sp. n. spherasters are always absent.

### Ecology and distribution

*Placospongia melobesioides* and *Placospongia mixta* are common in the reef environment. Most of the collected material from the reefs in Indonesia were one of these two species. *Placospongia carinata* appears to be rare in the reefs, in Indonesia at least, while it is highly abundant in the marine lakes Haji Buang and Kakaban in East Kalimantan, Indonesia. *Placospongia santodomingoae* n.sp. is restricted to an anchialine pool. *Placospongia anthosigma* was not found in any of the examined collections from the tropical Western Pacific, this species is restricted to more temperate and deeper waters. *Placospongia melobesioides* is indicated in the Systema Porifera to have a distribution from the Indo-West Pacific to the Tropical Atlantic (Rützler, 2002). Both *Placospongia melobesioides* and *Placospongia carinata* have been recorded from the Atlantic (e.g. de Laubenfels 1936, Hechtel 1976, Coelho and Mello-Leitão 1978, Pulitzer-Finali 1986, González-Farías 1989), which would imply that these are pantropical species. Recent molecular and more detailed morphological studies have, however, shown that many cosmopolitan sponge species are in fact species complexes either delineated by morphology or molecules (e.g. Reveilleud et al. 2010, Xavier et al. 2010). Van Soest (2009) has indicated that there are at least five species of *Placospongia* in the Caribbean that are morphologically different from the holotypes of *Placospongia melobesioides* and *Placospongia carinata*.Rua et al. (2006) and Nichols and Barnes (2005), furthermore, show that there are distinct lineages in the Caribbean and Western Pacific, that are not shared between the two regions and that most likely represent undescribed species in the Caribbean. Considering these results as well as the large geographic distance between the Caribbean and the type localities of *Placospongia melobesioides* and *Placospongia carinata* (both Indo-West Pacific), it is highly unlikely that these species occur in the Tropical Atlantic. Further revision of the Atlantic and Eastern Pacific material will shed more light on this issue.

Future biodiversity surveys and species checklists both in the Atlantic as well as in the Pacific are advised to check the spicule morphology of *Placospongia* specimens in order to identify species, as the external morphology and color will not give an indication to the number of species. The different *Placospongia* spp. can occupy the same type of habitats in the tropics. An example of such sympatry is represented in Kakaban lake where in the 4 km^2^ area of the marine lake the three common tropical species of *Placospongia* co-exist side by side. Neglecting to review the spicule morphology would mean possibly missing the true diversity of species that are present in the location of study.

## Supplementary Material

XML Treatment for Placospongia

XML Treatment for Placospongia anthosigma

XML Treatment for Placospongia carinata

XML Treatment for Placospongia melobesioides

XML Treatment for Placospongia mixta

XML Treatment for Placospongia santodomingoae

XML Treatment for Geodia labyrinthica
